# Persistent Habitat Instability and Patchiness, Sexual Attraction, Founder Events, Drift and Selection: A Recipe for Rapid Diversification of Orchids

**DOI:** 10.3390/plants14081193

**Published:** 2025-04-11

**Authors:** James D. Ackerman, Raymond L. Tremblay, Tatiana Arias, Gerhard Zotz, Jyotsna Sharma, Gerardo A. Salazar, Jaspreet Kaur

**Affiliations:** 1 Department of Biology, University of Puerto Rico, 17 Avenida Universidad Suite 1701, San Juan, PR 00925, USA; raymond.tremblay@gmail.com; 2 Department of Biology, University of Puerto Rico at Humacao, 100 Carr. 908, Humacao, PR 00791, USA; 3 Orchids for Peace, Sabaneta 055450, Antioquia, Colombia; tatianaarias@orchidsforpeace.org; 4 Functional Ecology Group, Institute of Biology and Environmental Sciences, Carl von Ossietzky Universität, D-26111 Oldenburg, Germany; gerhard.zotz@uni-oldenburg.de; 5 Smithsonian Tropical Research Institute, Panama City 08430, Panama; 6 Department of Plant and Soil Science, Texas Tech University, Lubbock, TX 79409, USA; jyotsna.sharma@ttu.edu; 7 Instituto de Biología, Departamento de Botánica, Universidad Nacional Autónoma de México, Mexico City 04510, Mexico; gasc@ib.unam.mx; 8 Department of Biology, University of Wisconsin, La Crosse, WI 54601, USA; jkaur@uwlax.edu

**Keywords:** Orchidaceae, habitat instability, epiphytism, mycorrhizal fungi, dispersal, founder events, drift–selection model, diversification rates, species richness, recent orogenesis

## Abstract

Orchidaceae is one of the most species-rich families of flowering plants, with most current diversity having evolved within the last 5 My. Patterns associated with species richness and rapid diversification have been identified but have not often been associated with evolutionary processes. We review the most frequently identified correlates of diversity and suggest that the processes and rate by which they occur vary geographically and are largely dependent on persistent pulses of habitat instabilities, especially for epiphytes. Aggressive orogenesis creates fragmented habitats while global climatic cycles exacerbate the ecological instabilities. The need for repeated cycles of dispersal results in frequent founder events, which sets the stage for allopatric diversification via bouts of genetic drift and natural selection. The allopatry requirement can be bypassed by pollination systems involving flowers attracting pollinators through the production of sex signaling semiochemicals. The drift–selection model of diversification, coupled with persistent habitat instability throughout ecological and geological time scales, and sex signaling are the likely components of a multifactorial process leading to the rapid, recent diversification in this family.

## 1. Introduction

Global patterns of biological diversity are well described for many plant families, and recent phylogenomic studies have revealed geographic variation in species richness that are not always matched with high diversification rates. These intriguing patterns beg for more process-based inferences [1], which are the population dynamics that affect natural selection and genetic drift [2]. The relative importance of selection and drift has formed the basis for models of evolution such as shifting balance, founder-flush, and genetic transilience [3,4] These processes are dependent on both intrinsic and extrinsic conditions, such as variation in genomic and epigenetic dynamics, effective population size, gene flow, dispersion, dispersal, aspects of reproductive biology, lifespan dispersion, and ecological interactions, among others.

The Orchidaceae is an intriguing family because it is cosmopolitan and extraordinarily species rich, representing approximately 10% of angiosperm taxa [5]. Furthermore, the high diversity and diversification rates differ geographically, and these are not necessarily correlated [6,7,8,9]. Here we propose that the process for rapidly diversifying Orchidaceae involves an interplay of natural selection and genetic drift, where conditions of persistent habitat instability occur and, to a lesser extent, where pollinator attraction involves sexual signaling.

## 2. Models of Diversification

Orchids have intrigued mankind since ancient times, and by the mid-19th century, orchid fever had begun in earnest, holding a special place in the consciousness of horticulturalists and botanists [10]. During that time when globalization was accelerating, explorers and plant collectors were sending shipments of spectacular orchids from the tropics to hothouses of Europe. Darwin [11,12] also contributed to this intrigue by launching the family as a prime example in support of his evolutionary studies. He interpreted the remarkable floral morphology of orchids as adaptations to increase the probability of cross-fertilization, an assumed generator of variation and a cornerstone to his theory of natural selection and descent with modification. In fact, orchid floral traits have repeatedly been shown to enhance the likelihood of cross-pollination (e.g., [13,14,15,16]).

Natural selection on traits that favor cross-pollination is clearly an important process, but it does not provide the full answer to why orchids are so diverse, because all sexually reproducing plant groups are subject to natural selection for traits associated with out-crossing, and clearly not all plant families are as diverse as orchids. The stock answer to the orchid species richness question became “adaptive radiation to different pollinators” [17,18]. An extraordinary example supporting this notion is *Disa* (Orchidoideae: Orchideae: Disinae), a genus primarily found in South Africa whose species specialize on a variety of functional groups of Diptera, Hymenoptera, and Lepidoptera [19,20]. Such specialization is likely the consequence of persistent selection for morphological matching to the most effective pollinator [18]. However, Johnson et al. [19] recognized that the extent of adaptive radiation observed in *Disa* is atypical and noted that related orchid groups in other geographical regions tend to be more conservative in their mode of pollination. Indeed, the diversity of pollinators of the genus *Disa* is on par with what we see at the subfamily level [21,22].

Orchidaceae is an old family with origins in the late Cretaceous (ca. 90 Mya), which has provided ample time for diversification at a Darwinian pace (gradual incremental changes). Nonetheless, much current diversity has developed quite recently, within the last five million years or less (e.g., [6,8,23,24,25,26,27]), suggesting alternative processes associated with diversification are at play.

One of the most orchid-rich regions of the world is the northern Andes [28]. Gentry and Dodson [29] suggested that the extraordinary diversity of epiphytic orchids in the Neotropics is likely generated by processes akin to Wright’s [30] “shifting balance” and Templeton’s [31] “genetic transilience” theories of evolution. Under these scenarios, multiple founder events play a major role in the interplay between drift and selection, generating “rapid evolution in a dynamic and kaleidoscopically changing habitat”.

Soon afterwards, Zimmerman and Aide [32], unaware of Gentry and Dodson’s paper, also suggested diversification processes are likely akin to Wright’s shifting balance theory of evolution, which considers that populations are divided into demes with small effective population sizes (Ne) and rare and inconsistent gene flow among them. To be evolutionarily isolated, gene flow among populations needs to be less than 1 effective migrant per generation when Ne is large (Ne > 50; [33,34]), and, if Ne is small (Ne < 50), then the effective number of migrants needs to be 1 or larger per generation [35] for natural selection to dominate genetic drift. With limited gene flow, subpopulations would evolve somewhat independently from each other with changes in phenotypic and genetic variation via genetic drift, followed by natural selection. Zimmerman and Aide [32] noted that founder events in combination with relatively short lifespans, variance in reproductive success, and biparental seed crops would exacerbate small Ne, likely making genetic drift an important component to the process of epiphytic orchid diversification. This process may be more common within a metapopulation context [36], characteristics of which are known for orchid populations [37,38,39,40,41,42,43,44,45]. None of the previous metapopulation-based approaches evaluated processes that are linked to Wright’s shifting balance or Templeton’s genetic transilience theories [4].

In one of the first attempts to evaluate the likelihood of genetic drift in orchid populations, Tremblay [46] explored the relationship between morphological variation within and among small populations of *Lepanthes* and suggested that restricted gene flow among populations is likely one of the causes of phenotypic dispersion. He showed that selection coefficients would need to be quite large (s > 0.20) to overcome drift. In general, selection coefficients have been found to be much smaller (example: [47]). A later population genetics study of three species of *Lepanthes* reaffirmed the conclusions of the morphological work. In most cases, Ne was less than 40% of standing populations regardless of the mathematical methods for estimating Ne [2]. Several other studies of orchid population genetics have measured Ne, often revealing very small effective population sizes, but not always (e.g., [48,49,50,51,52]).

Tremblay et al. [53] elaborated on the drift–selection (D-S) model with a comprehensive review of pertinent population data, much of which were consistent with the expectations of the model (e.g., dispersed populations, small Ne, low gene flow, founder events, low fruit set, and skewed reproductive success). Results from subsequent research testing expectations of the D-S model have been equivocal. Up through 2023, 749 articles cited Tremblay et al. [53], but only 25 specifically addressed the D-S model and 10 failed to find supporting evidence (data from https://scholar.google.com/scholar?oi=bibs&hl=en&cites=6232565937177585706&as_sdt=5, accessed on 15 January 2024), including a review of orchid population genetic data [54]). These results do not refute the importance of the D-S process but suggest that there may be regional and taxonomical differences in evolutionary patterns and processes [55,56].

### 2.1. Proposed Conditions Driving the Evolution of Orchid Diversity

While there is scant literature on evolutionary processes that may stimulate the rapid diversification in orchids, specialists on the family have long suggested reasons why it is so species rich, identifying practically everything that characterizes orchids as possible drivers of diversification (e.g., [57]). This has given us the following unhelpful conclusion: *orchids are diverse because they are orchids*. Only in the last few decades have correlates of diversity been re-examined within a phylogenetic context. Now, the most common approach to discover the underlying reasons for the extraordinarily high species diversity in the family is to look for geographical and functional patterns of diversity. Still, candidate drivers commonly associated with orchid diversity remain numerous (Table 1). We briefly review the literature on these drivers and look for patterns that are associated with high orchid diversity and diversification rates, but we also delve into the possibility that processes involved are context-dependent. We ask, where are orchids most diverse and diversification rates high? What factors are associated with high orchid diversification rates? And, finally, which geological, ecological, and evolutionary processes are congruent with the patterns revealed?

### 2.2. Where Are Orchids Most Diverse and Have High Rates of Diversification?

**Figure 1:**
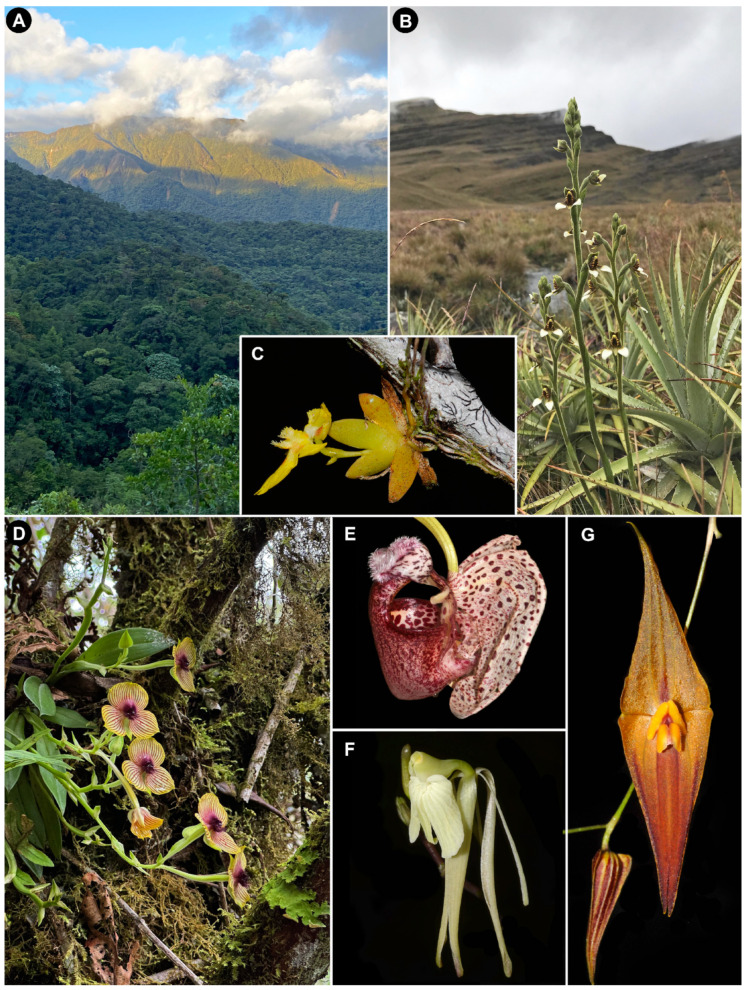
Northern Andes, a region of high species richness and high diversification rates. (**A**) Eastern Andean Ridge in northern Peru, where the Andes and the Amazon meet. (**B**) Terrestrial *Pterichis macroptera* flowering among bunch grasses and rosettes of *Puya* (Bromeliaceae) in a high-Andean grassland (jalca), northern Peru. (**C**) *Erycina glossomystax*, a “fast cycler” twig epiphyte; the thickest part of the branch is 5 mm in diameter; Ecuadorian Amazon. (**D**) *Telipogon* sp., a sexually deceptive member of the Oncidiinae; cloud forest near Oxapampa, central Peru. (**E**) *Coryanthes alborosea*, pollinated by perfume-seeking male euglossine bees; Peruvian Amazon. (**F**) *Epidendrum atonum*, a member of one of the most species-rich orchid genera worldwide; southern Ecuador. (**G**) *Lepanthes pastoensis*, representative of the extremely diverse and rapidly evolving subtribe Pleurothallidinae; northern Ecuador. All photographs by Gerardo A. Salazar.

A global model for plant diversity reveals the following two major species diversity hotspots: Neotropics and Southeast Asia Islands—Melanesia [79]. Patterns of high orchid species richness are quite similar. The simplest answer to where orchids are most diverse is the tropics, but not uniformly so. Dressler [28] mapped out orchid global diversity by continental region (and by country for the Western Hemisphere) when he estimated that there were about 19,000 orchid species. More than 40 years later, there are more than 33,000 recognized species, an increase of about 14,000 species [80] most of which reside in the tropics. The most recent study of global orchid diversity finds hotspots scattered through the tropics, primarily New Guinea, SE Asia, Madagascar, Atlantic Forest of Brazil, the northern Andes, and Mesoamerica [8]. Hotspots particular to Orchidoideae occur in temperate/subtropical regions of the Mediterranean, South Africa, New Zealand, and SW and SE Australia [9]. While some of these orchid hotspots are geologically ancient, the most species-rich regions are orographically recent mountainous areas in the tropics (Figure 1A; [8]).

Regions of high diversity are not necessarily the same as regions of high tip diversification rates. Thompson et al. [9] studied the geographical patterns of diversity and diversification rates for the Orchidoideae, the second largest subfamily of orchids (ca. 5000 species; Epidendroideae ca. 22,000 species [81]). Nearly all species of Orchidoideae are terrestrial, and the regions of high tip diversification rates are New Zealand, Australia, and southern Europe. Noteworthy is the observation that the high species diversity seen in southern Mexico and South Africa is not the product of recent evolutionary diversification. In contrast, the family wide analyses of Pérez-Escobar et al. [8] show that the highest diversification rates are from southern Mexico to tropical South America, with secondary regions of high diversification rates occurring in southern Asia and parts of Melanesia and Madagascar. The differences between the two studies may be due to the heavy representation of the largely epiphytic Epidendroideae in tropical regions, variation in sampling densities, and methodological differences (Pérez-Escobar personal communication, August 2024).

### 2.3. What Patterns of Orchid Natural History Are Related to Diversification?

Speciation requires barriers that limit gene flow amongst populations. One might expect that, in species-rich families, the mechanisms for reproductive isolation would be common. This might be expressed as specificity for the more obvious orchid symbionts, pollinators and mycorrhizal fungi. Is the specificity in these interactions associated with species richness?

#### 2.3.1. Specificity for Pollinators

The first symbiosis with a direct connection to reproductive isolation and gene flow involves plant–pollinator interactions. While adaptive radiation to different pollinator groups may not be as dramatic as that of *Disa* for orchids in general, selection for traits that are associated with the most effective pollinator may certainly provide reproductive isolation and maintain species integrity, allowing for the coexistence of related species. Specificity for pollinators is a hallmark of orchids across all subfamilies [22].

**Figure 2:**
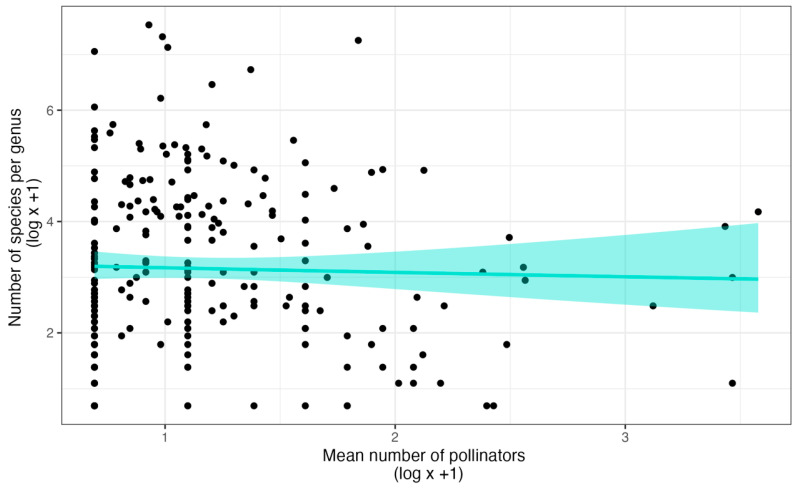
The relationship between specificity for pollinators and species richness of genera. The model represents the Gamma regression and the 95% confidence intervals (shaded area), the results of which are statistically indistinguishable from that of a linear model or LOESS regression. The dataset is from the supporting information of Ackerman et al. [22].

Nonetheless, it is not associated with species richness at the genus level. Gravendeel et al. [61] used data from van der Cingel [82,83] and found no association between the specificity and species richness of genera. Thus, specificity for pollinator services by itself may not drive speciation in orchids. We have recently confirmed this conclusion with a substantially larger dataset [22] (Figure 2). Of course, reproductive isolation by having different pollinators among closely related sympatric species does not require specificity, just different pollinator pools [84]. On the other hand, the median number of pollinators per species is less than two across all subfamilies, so there may not be enough variation to detect a relationship between species richness and pollinator specificity at either the subfamily or family levels.

The pollination system can make a difference in the levels of specificity that bely the lack of pattern between specificity and diversity across all genera. Specificity is particularly high in orchids that are pollinated via sexual deceit (Figure 1D). When sexual deception involves floral fragrances that mimic the sex pheromones of their pollinators, specificity is higher than any other type of pollinator attraction [22] and some of the genera involved are quite rich in taxa (*Pterostylis* 300 species, *Caladenia* 306 species, *Ophrys* 150 species and hybrids, *Telipogon* 252 species, *Lepanthes* 1203 species [80]). Rapid shifts in pollinator attraction may occur via minor changes in the fragrance composition, thereby increasing the diversification rates through evolutionary processes that do not require allopatry [85,86,87].

Similarly, the sexual attraction of pollinators *not* involving deceit may also have high diversification rates. Neotropical male orchid bees (Apidae: Euglossini) are attracted and rewarded by floral fragrances. The bees collect volatile compounds, and, in that process, they pollinate the flowers. The males then use the bouquet as part of their display to attract females [88]. As in sex deception, specificity tends to be high, but there is variation [89,90] which may be dampened as we engage in more sophisticated assessments of orchid variation and pollinator interactions that may reveal cryptic species [91]. As in sex deceit systems, a minor change in the fragrance composition can instantly alter the pollinator pool that is attracted [92,93]. More than 1000 Neotropical orchid species are pollinated in this fashion (estimated from Ackerman et al. [22], supporting information; Figure 1E), and, indeed, Givnish et al. [6] identified this pollination system as a significant driver of the high diversification rates in orchids.

#### 2.3.2. Hybridization

Interspecific hybridization is common in flowering plants and has been recognized as a means by which speciation may occur by generating unique genetic combinations on which natural selection may operate [94,95]. Ironically, despite the remarkable array of pollination mechanisms and the high levels of specificity for pollinators in orchids, interspecific hybridization is common in orchids. Wang et al. [96] found ample evidence that the breakdown of reproductive barriers has been a common phenomenon in the phylogenetic history of orchids. In fact, orchids have one of the highest propensities for hybridization among angiosperms, although, among orchid floras, it varies considerably [62,97]. While natural interspecific hybrids are known to occur in many genera, the frequency of hybridization can be quite low, hybrids can be sterile, and introgression has not often been reported (e.g., [98,99,100,101,102]). Furthermore, it is not clear whether there is a familywide connection between the frequency of hybridization and the high tip diversification rates. Nonetheless, hybridization is common in some species-rich genera with high diversification rates, including the pseudocopulated *Ophrys* and *Caladenia* mentioned above [79,103,104,105,106,107].

#### 2.3.3. Obligate Mycorrhizal Associations

The second important symbiosis involves orchid mycorrhizal fungi (OMF). Although Taylor et al. [108] had suggested that the diversification and specialization of orchids with their OMF have contributed to the rapid radiation of Orchidaceae, more recent literature indicates that there may not be a clear pattern. Mycorrhizal associations can alleviate intraspecific competition and enhance coexistence between host species, thereby enhancing diversity in plant communities [109]. However, whether mycorrhizal associations contribute to ecological speciation in plants [110,111] remains poorly understood. And while it is plausible that mycorrhizal fungi steer the evolutionary trajectories of their host plants by conferring adaptive traits, evidence linking host plant divergence to mycorrhizal communities is largely lacking.

Otero and Flanagan [64] argued that the obligate relationship with OMF for seed germination likely influences the spatial distribution of orchids which are often characterized by small, hyperdispersed populations whose individuals may be clumped or randomly distributed, particularly for epiphytic species [37,46,67,111,112,113,114]. Considering the multifaceted roles of mycorrhizal fungi in plant growth and development, and especially the requirement of OMF during orchid seed germination, it is conceivable that the distributions of mycorrhizal fungi shape the distribution of their host orchids [67]. The natural spatial distribution of OMF (and all fungi in general), however, remains poorly understood, which limits our understanding of the role of mycorrhizal fungi in driving the distributions, diversification, and ecological speciation of orchids [110,115].

In studies focusing on terrestrial orchids, evidence for the patchy distributions of OMF exists where the fungi themselves may have very limited distributions occurring only where the orchids occur [116,117]. Simultaneously, OMF can be locally abundant or widely distributed (e.g., [118,119,120]). Emerging evidence also indicates that fungi, including those that can form OMF, in tropical tree canopies have patchy distributions [67]. The patchiness of orchids may be affected largely by conditions affecting their OMF. In the case of terrestrial orchids, aboveground spatial patterns are governed, at least in part, by the belowground spatial distribution of their OMF, which can be very patchy [121]. Furthermore, OMF patchiness may be driven by local edaphic, ephemeral conditions (e.g., dead wood for wood-rotting OMF), making seed germination potentially patchy in space and time [66,122,123]. As for epiphytic orchids, some are more likely to occur on substrates covered with cryptogams, which may buffer fluctuations in water availability or the effects of toxic bark leachates, thereby benefiting not only the orchids but also their OMF [124,125,126]. On the other hand, OMF may be locally common and stimulate germination on a wide variety of phorophytes, but conditions for further development may exist on just a fraction of those phorophytes, thereby increasing the patchiness of adult plants [67,127].

Specificity for OMF may occur at the local population level or at the level of species regardless of geography. Sometimes an orchid may be specific for OMF locally, but more of a generalist over its distributional range. Kartzinel et al. [128] found that, within populations, peloton-forming OMF of the rare *Epidendrum firmum* were similar, but, among populations, the OMF were quite different, suggesting that, despite being rare, the orchid has the potential to associate with a range of OMF. In a contrasting case, where the specificity for OMF was compared between two common twig epiphytes which often grew together, one was highly specific (*Ionopsis utricularioides*) and the other was more of a generalist (*Tolumnia variegata*) [129]. Different epiphytic orchid species growing on the same branches may share the same OMF. Petrolli et al. [130] found that adjacent epiphytic orchid species on La Réunion Island utilize the same OMF, resulting in modular networks of orchids and fungi. In fact, the rapid diversification of orchids may occur independently of OMF diversity. Suárez et al. [131] examined the OMF of eleven sympatric, epiphytic *Teagueia* species (Pleurothallidinae) in the orchid-rich Andes of Ecuador, and found that they all shared the same OMF, so diversification, at least in this case, is related to factors other than specificity for OMF. Consequently, orchid–OMF relationships cover a plethora of possibilities, from being specific and rare, specific and common, to being generalist and common or generalist and rare. Furthermore, niche partitioning may occur as well as niche sharing (e.g., [132]).

Despite the known effects of mycorrhizal fungi on plant species coexistence [133], rarity [116,117,134], and adaptations [115], we lack a synthetic understanding of whether and how mycorrhizal fungi have shaped host plant radiation. Both ectomycorrhizae and arbuscular mycorrhizae show some indications of ‘non-independent’ evolution between host plants and mycorrhizal fungi [135], but similar information for orchids and OMF is not currently available. Mycorrhizal fungi are known to affect the reproductive traits of host plants by affecting the flowering time, duration of flowering, or number of flowers and fruits [136,137]. In fact, association with different mycorrhizal fungi resulted in distinct flowering phenologies for the same host plant [138,139]. This suggests that individuals of the same host plant associating with different mycorrhizal fungal partners could exhibit reproductive isolation within a population or among different populations. This reproductive isolation might steer ecological speciation within or among plant populations. However, so far, geography and pollination syndromes are typically implicated in driving reproductive isolation in plants. The only reports where mutualists are shown to affect speciation is in insects, where their symbiotic bacteria are known to cause reproductive isolation by changing the mating preferences and hybrid lethality of their hosts [140,141]. Whether mycorrhizal fungi can exert similar ecological interactions on plant reproduction and eventually on evolutionary processes currently remains unknown.

Thus, the evidence for orchid–OMF specificity driving speciation is equivocal at best. In fact, Givnish et al. [6] noted that mycoheterotrophic seed germination was not by itself a driver of diversification. Nonetheless, OMF are patchily distributed, so that the combination of these symbioses (pollination and mycorrhizal) may affect the population size, spatial distribution, and gene flow, all of which may affect speciation [53,56,64,122].

### 2.4. Reproductive Morphology

Most orchids are severely pollination-limited, either because pollinators are scarce, they lack rewards attracting only naïve pollinators, or populations represent a resource too meager to generate pollinator return visits [53,142]. Under these circumstances, selection may favor an autonomous self-pollination system. In fact, up to 19% of orchid species are autogamous at least in some populations, a condition that may be an evolutionary dead end, but not necessarily so [22,143,144]. Regardless of these limitations, orchids exist, and by the criterion of species richness, they are one of the most successful flowering plant families. Flower biology makes the difference. Pollinators are guided to a position where pollen deposition and removal is consistent. In most subfamilies, pollen is packed into discrete units (pollinia), most, if not all, of which is deposited on a stigma in a subsequent visit to another flower (especially Epidendroideae). This pollination event will generally deliver sufficient pollen to fertilize perhaps all ovules and can result in biparental seed crops [53]. Although rarely studied, multi-paternal pollinations are likely rare in orchids because of infrequent pollinator visits [145,146,147,148]. In most orchids, ovules do not develop until after pollination [149]. The number of seeds in a fruit may be less than 100 in some of the smallest fruits to several million in species with the largest fruits [150,151]. Consequently, DNA recombination during sporogenesis can contribute substantially to genetic diversity within a single orchid fruit. This abundance of offspring provides ample material on which evolutionary mechanisms may operate, potentially leading to a diverse array of adaptations.

Seeds of orchids are dust-like and wind-dispersed with very few exceptions [150,152,153,154]. Variation in seed micromorphology among orchid species is generally constrained phylogenetically [155], but some seed traits are associated with latitude and habit (terrestrials and epiphytes) [156,157]. For example, hooks on the seeds of twig epiphytes may help with attachment to substrates [158]. Epiphytes have larger embryos and less airspace between the testa and embryo, which may lower the buoyancy [159,160]. The ecological significance of these micromorphological patterns is not always clear. However, in field seed trap experiments of wind-dispersed epiphytic and terrestrial orchids, most seeds fall near parents [161,162], yet laboratory data indicate that the height above ground is positively associated with the probability of long-distance seed dispersal, and the higher the wind speed, the greater the variability in dispersion distances [163]. Small, distant populations may become established with a low likelihood of additional gene flow to or from the parental population. The extraordinary number of minute seeds produced in a single fruit not only represents an equal number of genetic combinations but also increases the probability of landing on a branch, as well as experiencing long-distance dispersal. Successful long-distance dispersal need not be common to be effective, and there are many cases in which it is inferred. For example, populations of epiphytic *Epidendrum mutelianum*, a cloud forest inhabitant in Puerto Rico, is 300 km downwind and across the Caribbean Sea from the nearest possible source in the Lesser Antilles. Others temporarily survive as waifs, such as the rupicolous/epiphytic *Ida pegueroi* in Puerto Rico, >350 km from the closest possible source. More dramatically, the rare endemic terrestrial, *Anoectochilus sandvicensis* of Hawai’i, is found >6000 km away from its congeners (based on POWO [80] distribution maps and the Google Earth Pro version 7.3.6.10201 distance-measuring function). The consequences of these dispersal capabilities at local to regional scales are that epiphytic and terrestrial orchid populations usually appear to be small, locally aggregated, and randomly or hyperdispersed [37,46,111,113,114,164]

### 2.5. Vegetative Morphology

Besides the remarkable diversity of orchid flowers, the other striking aspect of the family is the morphological diversity of the roots, stems, and leaves. Plants can be geophytes, vines, or epiphytes; deciduous, evergreen, or leafless. They can have monopodial or sympodial growth and even a combination of the two (e.g., *Dichaea*). They may be epiphytes with photosynthetic roots growing on tree trunks or twigs. Stems may be wiry or succulent, and leaves may be delicate, tough or succulent, plicate or conduplicate, or even absent [28]. Such diversity indicates the multiple ways an orchid can occupy soil, rocks, and trees, which increases opportunities for diversification.

Stem and leaf succulence, often associated with the crassulacean acid metabolism (CAM), preceded the ascent to the epiphytic habitat and is inferred to have evolved in a terrestrial ancestor more than 43 million years ago. Just four million years later, orchids started to conquer the epiphytic habitat; epiphytism now exists in four of the five subfamilies [69]. Only the earliest diverging and smallest subfamily, Apostasioideae, lacks both succulence and epiphytism in extant species. Nonetheless, the transition from a terrestrial to an epiphytic habit for orchids or other plants is not at all contingent on succulence, particularly in humid montane habitats [165]. Silvera et al. [70], Collobert et al. [69] and others have linked both succulence and CAM to epiphytism in orchids, but such a link is doubtful [166,167,168].

### 2.6. Physiology

All orchids are heterotrophs during germination (dependent on exploiting OMF) and some remain so throughout their life. However, by far most become either autotrophs or, in a few cases, mixotrophs (autotrophs, yet still gaining some nutrition from fungi). Photosynthesis can be either C3 or CAM; there are no known cases of C4 photosynthesis in orchids. While C3 photosynthesis is considered ancestral in the family, CAM has evolved independently in numerous lineages of orchids, including four of the five subfamilies, most commonly in the Epidendroideae, even in leafless epiphytic orchids, which are entirely dependent on root photosynthesis [70,169,170]. The relative prevalence of CAM, its ecological importance, and its role in the speciation of epiphytic orchids are a matter of considerable interest and debate. For example, while earlier estimates of the number of epiphytic orchids with CAM were as high as 50% [171], later extensive surveys have yielded figures of around 10% [167,172]. This also challenged our view of its ecological role. The relative proportion of CAM species hardly differs between epiphytic and terrestrial taxa in the extensive survey of Colombian orchids of Torres-Morales et al. [166]: epiphytes: 9.5%, or 76 of 805 species; terrestrials: 7.3%, or 19 of 260 species. Lastly, while some studies have reported associations of the presence of CAM with high speciation rates in the family [6,70], the results of studies with the extraordinarily species-rich genus *Bulbophyllum* disagree (c. 2340 species [80]). Among Malagasy *Bulbophyllum* species, CAM had no significant effect on diversification rates, yet may have provided a gateway that considerably broadened the spatio-ecological amplitude of the genus, as has been suggested for its sister genus, *Dendrobium* [173,174]. In another study, *Bulbophyllum* of Southeast Asia showed high speciation rates associated with CAM, but also high extinction rates, which effectively dampens the role of CAM as a key innovation in the net diversification of the genus [175].

### 2.7. Genomic Architecture

Orchidaceae has the greatest genome size diversity among Angiosperms and its subfamilies have distinct genome size profiles [71,72,176]. The largest genome sizes are in the early-diverging subfamilies Cypripedioideae and Vanilloideae of terrestrial habits, whereas the Epidendroideae, the largest, predominately epiphytic subfamily, has the greatest range in genome sizes, which is skewed to small genomes [72,176]. While the range of genome sizes of terrestrial and epiphytic species overlap, the distribution is bimodal, with terrestrial species in subtribe Oncidiinae having larger genomes than epiphytic species [71]. Consistent differences between epiphytic and terrestrial species have also been reported for Araceae [177]. Genome sizes are positively correlated with the guard cell size and negatively correlated with the stomatal density (in orchids and in general), which may be adaptive under the low-nutrient and water-stress conditions that epiphytes experience [71,178]. Either small genome sizes have predisposed lineages to become epiphytic, or the epiphytic habit imposed strong selection for smaller genomes (less nutrients needed for genome maintenance and faster cell cycle to take advantage of sporadic water availability), smaller guard cells, and higher stomatal densities (better evapotranspiration control) [71,176,179].

Across angiosperms, waves of genome expansion and contraction are not only associated with the evolution of anatomical and physiological traits [180] but are also linked to speciation rates [181]. For orchid clades that have high diversification rates, this relationship is not clear. For example, the species-rich genera of Pleurothallidinae, such as *Lepanthes, Pleurothallis, Stelis, and Masdevallia*, all have very small but variable genome sizes, yet possess high diversification rates [8,182], suggesting that either speciation is occurring independently of changes in genome size, increases are rapidly followed by decreases that would be difficult to capture, or the genome size changes associated with speciation rates do not need to be dramatic.

### 2.8. Epiphytism

All roads seem to lead to epiphytism: pollinia, dust seeds, succulence, CAM, and small genome sizes. Approximately 75% of orchid species are epiphytic [183], and epiphytic genera have significantly more species than terrestrial ones [61], suggesting that something about being an epiphyte facilitates diversification. Gentry and Dodson [29] proposed that one of the reasons why epiphytes are so diverse in the northern Andes is because of the high microsite diversity within trees offering the possibility for niche partitioning (viz. [184]). While microsite specialization is often thought to be driven by competitive interactions, there is very little evidence for interspecific competitive interactions among epiphytes [185,186], presumably because the substrate is not only inherently ephemeral, but also because microclimatic conditions on any part of the tree change as trees grow or senesce [73,187]. Nonetheless, selection to specialize on a particular microclimatic niche need not be driven by competition. For example, Tremblay et al. [188] showed that selection coefficients on morphological characteristics based on light availability within a phorophyte influences the reproductive potential.

Using a phylogenomic approach, Givnish et al. [6] found that orchid diversification rates were significantly higher for epiphytic taxa than terrestrials (Figure 1B–D). This is also true within genera. Epiphytic clades of *Paphiopedilum* have much higher diversification rates than those of terrestrial species [189], and clades of Dendrobiinae dominated by twig epiphytes with their small size and short life cycles were repeatedly correlated with bursts of speciation [61]. Years earlier, Gentry and Dodson [29] used anecdotes of population turnover in twig epiphytes of the genus *Comparettia* (formerly *Scelochilus* species) to illustrate how rapid orchid diversification may occur in response to environmental changes.

Stress acts as a primary stimulus that induces genetic changes in plants at multiple molecular levels, including genomic, regulatory, and epigenomic. It is generally assumed that epiphytic orchids face highly variable environmental conditions that impose unique physiological stresses. Orchids can respond to these stresses via phenotypic plasticity [190,191,192], but such responses will only lead to diversification when stress-induced plasticity modifies the genomic architecture of the organism. There are multiple ways by which this may occur [193]. For example, plasticity may promote diversification because the developmental pathways that underlie phenotypic plasticity consist of many genetic components, each one of which may vary and be subject to selection imposed by environmental stresses [194]. While the diversity and diversification rates of orchid epiphytes are high, rapid diversification can also occur in terrestrials [9], even taxa with large genomes such as *Cypripedium* [63,195].

Genomic stresses, including biotic or abiotic, can restructure epigenetic signals, triggering bursts of transposition, and producing a wide variety of changes in plant gene expression and function [196]. The propensity to reprogram gene expression is in itself advantageous, and transposon elements (TEs) represent a mechanism by which the genome may be poised to respond to stress by reorganizing itself [197,198]. Some examples of genetic changes caused by TEs include gene inactivation, the reprogramming of gene expression, rearrangements, and gene transpositions (gene capture).

Endoreplication is emerging as an important factor in the stress response of plants [199]. The influence of stress on the molecular evolution of metabolically active cells in orchids through endoreplication is a topic that has received limited investigation [176,182,200]. Nonetheless, it is conceivable that this mechanism serves as an adaptive response to exploit either intermittently favorable conditions, particularly pertinent for epiphytic orchids, or to adjust the nuclear to organelle ratio and to increase gene copy numbers to mitigate environmental damage. The precise role of endoreplication in providing an adaptive advantage for epiphytic orchids to withstand stressful conditions and potentially drive diversification rates in specific orchid groups remains to be fully elucidated.

Thus, there are mechanisms by which inheritable variation may be generated in response to stress. Here, we assume that the stresses experienced by an epiphyte generate more variation than the stresses faced by terrestrial species. With more variation, there may be more opportunities for selection and subsequent diversification. However, if we just consider epiphytes, there are large differences in diversity among regions. While orchids do occur in dry forests where physiological stresses are paramount, orchids in such habitats are not nearly as diverse as orchids in wetter ones. In fact, vegetation complexity, plant diversity, and diversification rates peak at mid-elevation cloud forests [201,202,203,204,205]. Fluctuations in water and nutrient availability for epiphytes in these habitats may be minimal [183], suggesting physiological stress is not a generator of diversity and may be a hindrance to rapid orchid diversification. *Regardless of the relationship between physiological stress and orchid diversification, we propose that factors related to population dynamics may be sufficient to explain why diversification rates are highest among orchid epiphytes. We address this in the following section.*

## 3. Correlates of Diversification

Globally, high tip diversification rates in orchids are associated with epiphytism in regions of rapid orogenesis. Nonetheless, the functional traits, ecological and geographical patterns of diversity, and diversification rates by themselves do not reveal the population processes involved in diversification. This leads us to ask: what are the evolutionary processes that are mainly responsible for speciation in the orchid family? We argue that these processes vary geographically and, where diversification rates are high and recent, multiple factors act in concert to push orchids from being an ordinary to an unusually species-rich family.

### 3.1. Persistent Instability

In regions where recent high diversification rates exist, persistent habitat instability occurs across expansive spatial and temporal scales [68]. At a generational time scale, abiotic and biotic conditions for epiphytes constantly change as trees grow, and bark, branches, and leaves are shed. For any location on a phorophyte where an orchid may become established, light intensity and quality, air movement, moisture and nutrient availability, and biotic interactions with microbes, lichens, bryophytes, and pollinators may change [206]. While phorophyte niches for orchid epiphytes are constantly in flux, the trees themselves are ephemeral, not only subject to aging, but also affected by extrinsic forces such as successional processes, droughts, wind storms, landslides, and lightning strikes, resulting in tree mortality rates of about 1% a year (e.g., [207,208,209,210]. Epiphytes would need to have the physiological and developmental plasticity to cope with microsite change (e.g., [211]).

**Figure 3:**
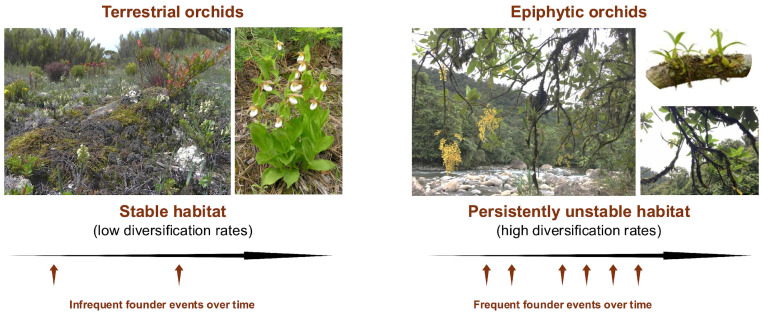
High instability and heterogeneity of the epiphytic habitat makes founding events for epiphytes more frequent, which provides for opportunities for selection and drift. The height of seed release makes long-distance seed dispersal more likely. Pollination systems involving sexual signaling can accelerate the diversification somewhat independently of founder event frequencies for both terrestrials and epiphytes.

Given the relative instability of the epiphytic habitat, dispersal capabilities are essential for the long-term survival of epiphytic orchid populations. As habitat conditions change, dispersal becomes a necessity, and orchids are quite adept at that, given their dust-like, wind-dispersed seeds. Locally, metapopulation dynamics are likely at play [37,40,212], but long-distance dispersal occasionally occurs, as evidenced by orchids being among the first terrestrial and epiphytic vascular plants to colonize Krakatau after the 1883 eruption [213]. Under these conditions of persistent instability and effective dispersal, epiphytic orchids are resilient, yet their populations are expected to be small, hyperdispersed, and relatively ephemeral (Figure 3). Because of the need to constantly establish new populations, population dynamics may mirror aspects of Wright’s shifting balance, Carson’s founder-flush, and Templeton’s genetic transilience models of speciation, where the persistent occurrence of founder events gives novel genetic combinations a greater chance of becoming established [4,214,215,216].

Habitat instability is not just about cycling through phorophytes, it is also prolonged in time and space. The richest regions of orchid diversity and high diversification rates are in the tropics, and associated with relatively recent and ongoing orogenesis, allowing cloud forests to establish where orchid species richness is so evident [8]. Most of these areas are active tectonically, subject to severe earthquakes and violent volcanism, with repeat occurrences on the scale of decades to thousands of years (e.g., [207,217,218,219].

The importance of recent orogenesis to orchid diversification is underscored by phylogenetic analyses. Species of upper elevations may be derived from more widespread lowland species or have arrived via natural dispersal from other mountainous areas, a question addressed by Wilson [220], Ricklefs and Bermingham [221], Merckx et al., [222], González-Orozco [223], and others, for a wide range of plant and animal taxa and regions. The orchid data are thus far relatively consistent. Barkman & Simpson [224] discovered that high-elevation *Dendrochilum* endemics of Mount Kinabalu (Sabah, Malaysia) were likely derived from lowland species of Borneo, not from the high-elevation ancestors of other areas of the region. Similarly, Kirby [225] noted that, in Costa Rica, older lineages of Maxillariinae were of the lowlands and more recently diverged species were residents of upper elevations. This general pattern is also seen for the tribe Cymbidieae and subtribe Pleurothallidinae of the northern Andes, but with some dispersal from high-elevation taxa of Central America [204].

### 3.2. Geographic Variation in Habitat Instabilities and Diversification Rates

The Cordillera de Talamanca of western Panama and southeastern Costa Rica, and the eastern cordilleras of the northern Andes are regions with extraordinarily high orchid species richness and diversification rates [8]. For the last 2.5 million years, orogenesis of the Talamanca range has occurred at a rate of approximately 1 mm/yr in elevation [226], creating a multitude of climates varying in temperature and precipitation. Similarly, over about the last 5 million years, the uplift in the Eastern Cordillera of Colombian Andes is estimated as varying from 0.2 to 3 mm/yr [205,227]. Other major geological and climatic repercussions were taking place in these regions of the Neotropics, including the formation of the Caribbean via the closure of the Isthmus of Panama, and the Amazon delta flow change from the Caribbean to the Atlantic in the Late Miocene ±2–7 Mya [228]. These interconnected events profoundly shaped the region’s geology, climate, and biodiversity [205,229,230].

A hotspot of high orchid species richness but moderate diversification rates is at the eastern escarpment of tropical Madagascar, the face of which rises about 1000 m [8,231,232]. In contrast to the Talamancas or the northern Andes, recent orogenesis in Madagascar is not involved. Instability is based on erosion. The escarpment has been moving westward *horizontally* at about the same rate as the orogenesis of the Talamancas, approximately 1 mm/yr (estimated from Liu Y et al. [232]). The difference is that new habitat types are not being created as orogenesis would. While the geological instability of the escarpment may be a factor in orchid diversification, the paleoclimatic variation in moisture regimes during the Pliocene to the Holocene [233,234], particularly the dry periods, likely hampered the diversification rates.

In contrast to tropical regions dominated by epiphytic Epidendroideae (Figure 1F,G), regions of high species richness of terrestrial Orchidoideae occur in non-tropical regions of southeastern and southwestern Australia, and South Africa [8,9]. All three are relatively stable geologically but have experienced gradual shifts or frequent oscillations in moisture and/or temperature regimes over the last 5–6 million years [235,236,237]. Among these regions, southeastern and southwestern Australia orchid floras also have high diversification rates according to Thompson et al. [9] with species of some clades of Pterostylidinae having diversified over the course of less than 0.5 Mya [76,77]. By contrast, the South African orchid flora, which is also rich in Orchidoideae, has low diversification rates according to the analyses of both Thompson et al. [9] and Pérez-Escobar et al. [8]. Why is this region so different from those in Australia? The answer may reside in the prevailing pollinator interactions of the respective regions, which may affect the diversification rates (e.g., [238]). In southern Australia, sexual deceit is the dominant mechanism of pollinator attraction for orchids (about 70% of the 205 species for which we have data [239]. Minor changes in sex pheromone-imitating floral odors may attract different pollinators and result in reproductive isolation and rapid evolution, likely a process that is much less dependent on founder events and allopatric speciation [84,85,86], whereas, in South Africa, sex deception is rare [240]. Another factor that must be considered is the propensity for hybridization. Among angiosperms, orchids have one of the highest weighted hybridization propensities, at 6%, whereas it is about 0.9% for the orchid flora of South Africa [62,97]. Thus, the combination of the low frequency of hybridization, habitat stability, and the lack of sex signaling in pollination systems of South Africa result in diversification that proceeds incrementally, at a Darwinian pace.

One might expect that tropical rainforests would be ideal for orchid diversification if located in regions of topographic diversity and recent orogenesis [8]. Equatorial Africa currently has relatively low measures of both orchid diversity and diversification rates [8,9]. This should not be surprising given that, as recently as the last glacial maximum (18,000 ybp), most of the region was lowland semideciduous forest (with a dry season of 2–4 months). Furthermore, in equatorial East Africa, which has mountainous topography, the vegetation was mostly grass savannas, dry forests with isolated pockets of montane forest [241]. These are not the conditions where one finds a high diversity of orchids [204,242].

### 3.3. Climate Instabilities and Diversification in Terrestrial Orchids

Oscillating climatic conditions may drive the diversification of terrestrial orchid clades, and these may be independent of rapid orogenesis. In a genomic study of *Ophrys sphegodes*, a sexually deceptive Mediterranean species, Russo et al. [78] found that long terminal repeat element insertions peaked 0.8–1.3 Ma when the region was subjected to climatic oscillations of glacial/interglacial periods. This also coincides with the period when the *sphegodes* clade diversified. Similarly, Lagou et al. [63] suggested that glacial cycles in the Late Pliocene and the Quaternary may have promoted the diversification of *Cypripedium* by isolating populations in refugia, and Liao et al. [189] offered that the uplift of the Qinghai-Tibetan Plateau occurring 6–2 Mya was the stimulus for the diversification of most *Cypripedium* taxa by providing isolated populations at cooler, higher elevation habitats.

Across the largely terrestrial subfamily Orchidoideae, Thompson et al. [9] revealed that the high diversification rates were most associated with historic global cooling over latitude, elevation, chromosome number, or other historic climate change variables. This includes the Mediterranean region, where, for example, most species of Orchidinae diversified since the mid-Pliocene [243]. The authors do not propose a mechanism for diversification based on climate cooling, but there are various mechanisms that might be at play. First, climate change can impose stresses in which the plants respond through the various genetic mechanisms mentioned earlier. Secondly, phenotypic plasticity can bring a multitude of alleles into play that are not normally expressed. These would be subjected to natural selection [194]. Thirdly, since phenology is temperature-dependent in cool climates (e.g., [244]), populations can become isolated depending on the initiation of growth and reproduction due to conditions such as aspect and exposure, leading to drift or opportunities for adaptation to local conditions [245,246] and, ultimately, speciation. Lastly, changing environmental conditions necessitate dispersal to reach more suitable habitats, resulting in repeated founder events and subsequent selection. While these are not mutually exclusive processes, a combination may accelerate speciation for terrestrial species. Similar to suggestions of Russo et al. [78], Lagou et al. [63], and Liao et al. [189], the result is a reduction in suitable habitats and an increase in population isolation, reducing gene flow and providing the opportunity for drift and selection to drive rapid diversification.

In contrast, Antonelli et al. [75] proposed that the rapid diversification of rupicolous *Hoffmannsegella* (Epidendroideae: Laeliinae) in Brazil occurred in the Middle/Late Miocene in response to climate cooling. Instead of habitat fragmentation driving speciation, previously isolated populations became connected. Vegetation shifted from forests to open savannas, connecting previously isolated species. The authors speculated that this contact resulted in hybridization, variably fit hybrids followed by genome duplication restoring fertility and generating new lineages.

### 3.4. Patterns and Processes

Given that instability is persistent at multiple spatial and temporal scales from a tree branch to substantial orogenesis, and from time spanning generations to millions of years, metapopulation dynamics are essential for long-term persistence. Suitable habitats are often ephemeral and isolated, fragmented over time by shifting physiography and climate. Orchids, with their dust-like seeds produced in prodigious quantities, are adept at coping with such conditions, but there are limitations, which may be related to the availability of OMF or simply physiographic barriers. In a study of Andean orchids of Colombia, one of the global hotspots for species richness and tip diversification rates, orchid dispersal appeared constrained and species turnover was more related to geographic distance than niche specialization [74].

In regions of high diversification rates, we expect that persistent habitat instability would make the relative frequency of small effective population sizes to be high largely because the fruit set is generally low, and seed crops are often biparental [53]. Consequently, successfully dispersed seeds involved in founder events should have a higher probability of being unusual, not entirely representative of parental populations, and may include unusual combinations, perhaps even hopeful monsters via mutations that affect the rather unique box genes for orchid floral morphology, known as the ‘orchid code’ [247,248]. Unusual variants may initially involve just minor differences that could have major consequences by attracting a novel set of pollinators by exploiting their sexual behaviors. This sets the stage for rapid speciation via the drift–selection processes described by Tremblay et al. [53]. Repeated founder events are key. Once established, populations can grow rapidly in the absence of competitive constraints, undergo genetic transilience (increased inbreeding without the loss of genetic variability), and experience selection intense enough to overcome the effects of drift in small populations, akin to the founder speciation model of Templeton [215]. Under this drift–selection scenario, significant change is likely spasmodic, not incremental.

Thus, for orchids, we suggest that persistent instability drives the repeated cycles of drift and selection, the probability of which is certainly context-dependent. While it may occur in the tropics as well as temperate regions, and in populations of terrestrials as well as epiphytes, it most likely occurs in small populations of epiphytes with little or no competition, across naturally fragmented mountain landscapes of recent orogenesis. Persistent instability in substrates, environmental conditions, regional climates, and geology occur over all time scales, making it imperative to be adept at founding new populations.

The patterns and processes involved in the diversification of the Orchidaceae are not necessarily expected to be mirrored by other species-rich plant taxa. As we have addressed here, drivers of diversification among orchids can vary by taxon, habit, pollination biology, geography, and historical geology. We certainly expect that other families diversify in different ways as well, since each family has its own unique suite of morphological and functional traits, natural histories, geography, and selection regimes. Indeed, diversification rates in species-rich families, such as Asteraceae, Bromeliaceae, Cyperaceae, Fabaceae, Poaceae, and Polypodiaceae, usually have multiple drivers acting in concert or separately in different clades that may change over time, including some of the same as reviewed here for orchids, such as epiphytism, pollination biology, climatic shifts, and orogenesis [165,249,250,251,252,253].

## 4. Future Directions

Over the last 25 years, our understanding of orchid evolution has expanded substantially, as we have outlined here. However, relative to the size of the family, our knowledge of the patterns and processes of orchid evolution is in dire need for more intensive taxon sampling across the planet to refine the areas and conditions of rapid diversification. Certainly, genomics can advance our understanding of orchid diversification by using high-throughput sequencing to resolve phylogenetic relationships within species-rich clades, such as Pleurothallidinae and Dendrobiinae, as well as genera that exhibit high morphological diversity. Genomic data would also allow us to better detect hybridization and introgression, processes that may be more common in orchids than previously thought, maybe even critical for rapid diversification. Whole-genome duplications across key lineages could reveal how polyploidy has contributed to species diversification and ecological adaptation. Last, integrating genomic tools with ecological data could help to unravel the genetic basis of adaptations to epiphytism and other niche specializations, shedding light on how these traits have driven orchid radiation. From the perspective of processes, we need a better understanding of orchid life-span variation and estimates of effective population size, especially in regions of high species richness and rapid diversification rates, to address the interplay between phenotypic selection and random processes (drift).

## 5. Conclusions

Orchids are resilient ecologically and evolutionarily but require particular life history conditions. Where orchids are both species rich and have high tip diversification rates, their habitats, especially those of epiphytes, experience persistent instability at all spatial and temporal scales, yet capture suitable habitats by their dispersal capabilities. This habitat instability drives metapopulation dynamics, requiring repeated founder events, while orogenesis, erosion, and climate oscillations create fragmented habitats that limit the homogenizing effect of gene flow, thereby powering the engine of diversification. These processes may be enhanced or accelerated by frequent hybridization and pollination systems involving sexual signaling via floral fragrances. Thus, we have overlaid the drift–selection model on a bed of persistent instability and flavored by geographical context variation. This is the *drift–selection and persistent instability process of rapid orchid diversification.*

## Figures and Tables

**Table 1:** Factors commonly associated with orchid species richness and diversification. Rationale listings are based on the literature. See the text sections for our assessments of each one.

Factors	Rationale	Select References
Tropics (Section 2.2)	Potential for uninterrupted growth; higher phorophyte diversity; higher functional diversity of pollinators; higher orchid mycorrhizal fungal (OMF) diversity; higher niche specialization; diverse array of spatial niches	[58,59,60]
Pollinator relationships (Section 2.3.1)	Remarkable adaptations for precision pollination; high specificity for pollinators; deception pollination; severe pollination limitation; sexual attraction based on floral odors	[6,17,22,55,61]
Hybridization (Section 2.3.2)	Instantly creates novel genetic combinations and novel phenotypes that may be subjected to selection	[62,63]
Mycorrhizal relationships (Section 2.3.3)	Obligate symbiosis with orchid mycorrhizal fungi (OMF); patchiness of OMF; specificity toward OMF	[64,65,66,67]
Wind-dispersed, dust-like seed (Section 2.4)	Seeds produced in large quantities; stochastic element to reproductive success; broadcast dispersal to reach suitable but patchy and ephemeral habitats	[53,68]
Pollinia (Section 2.4)	Single pollination event delivers massive amount of pollen to fertilize thousands of ovules; biparental seed crops result in extensive sampling of recombination possibilities, including rare alleles	[32]
Succulence (Section 2.5)	Provides water storage in habitats with sporadic water availabilities; predates epiphytism	[28,69]
Crassulacean acid metabolism (Section 2.6)	Provides water-efficient carbon fixation; presumed to be common among epiphytes	[6,70]
Genome size (Section 2.7)	The most diverse clades have small genomes and are associated with rapid cell cycling under intermittent water and nutrient availability	[71,72]
Epiphytism (Section 2.8 and Section 3.1)	Novel environment, open niche; greater surface area availability; microsite niche partitioning; the most common substrate for orchids; locally ephemeral, fragmented habitat; low levels of competition	[29,61,68,73]
Mountainous habitats (Section 3.2)	Fragmented landscapes lead to isolated populations; highly variable topography influences orographic precipitation and other abiotic conditions, providing barriers to dispersal	[29,74]
Climate change (Section 3.3)	Paleoclimate cooling associated with orchid speciation; increased variation in genome associated with climate fluctuations	[9,75,76,77,78]
